# Incidental Neoplasm and Histopathological Spectrum of Suspected Acute Appendicitis in Appendectomy Specimen: An Observational Study

**DOI:** 10.31729/jnma.8914

**Published:** 2025-03-31

**Authors:** Archana Tiwari, Pratima Sapkota, Dipesh Shrestha

**Affiliations:** 1 Department of Pathology, Lumbini Medical College, Palpa, Tansen, Nepal

**Keywords:** *appendicitis*, *appendix*, *carcinoid*, *histomorphology*, *incidental neoplasm*

## Abstract

**Introduction:**

Acute appendicitis is the most common surgical emergency and histopathological studies are the gold standard for confirmation of clinical diagnosis and key methods of discovering appendiceal neoplasm. Appendiceal neoplasms are uncommon and are mostly identified by pathologic examination after appendectomy for presumed appendicitis as an incidental finding.

**Methods:**

An observational cross-section study was conducted at a Medical College from 1^st^ August 2022 to 31^st^ July 2024. Appendectomy specimens were included in the study after obtaining consent. Ethical approval was taken from the Institutional Review Committee (Reference number: IRC-LMC 05/S-22). Descriptive analysis was done, frequency and proportion were calculated.

**Results:**

Among 350 appendectomy cases, 30 (8.57%) cases had negative appendectomies. Out of total 320 patients, 183 (57.19%) were male and 137 (42.81%) were female. Histopathological findings suggestive of acute appendicitis were observed in 164 (51.25%) specimens and there were 6 (1.87%) specimens suggestive of neoplasms. Amongst the neoplasm of appendix 3 (0.94%) were carcinoid tumours.

**Conclusions:**

Appendiceal neoplasms are uncommon which was comparable to previous studies. Carcinoid tumour was the most common incidental neoplasm.

## INTRODUCTION

Acute appendicitis is among the most common cause of acute abdomen,^1^ representing a significant burden at histopathology laboratory with frequent incidental neoplasms.^2^ The appendix is likely to become infected, ruptured, and gangrenous, causing peritonitis and even death. Consequently, Appendectomies are common specimens in histopathological labs for diagnosis and to establish etiology and associated findings. Appendiceal neoplasms manifest with obscure symptoms presenting as acute appendicitis, as seen in 30%-50%.^3^

The incidence of appendiceal tumours has increased from 0.12 to 0.97 per 100,000 populations according to a 2023 article.^4^ This increase reflects greater recognition and reporting. Appendiceal neoplasia represents < 1% of gastrointestinal neoplasms. Appendiceal Neuroendocrine Tumours (NETs) are often discovered incidentally due to their small size and indolent behaviour, but may manifest with nodal involvement or metastasis. Radiological imaging findings and symptoms of primary appendiceal tumours overlap with acute appendicitis.^5^ Therefore, this study aims to find the proportion of incidental neoplasm and histopathological spectrum of acute appendicitis in appendectomy specimens.

## METHODS

This is an observational cross-section study conducted at Department of Pathology, Lumbini Medical College Teaching Hospital (LMCTH), which is a tertiary care medical college situated at Lumbini province, Nepal. This study was conducted between 1^st^ August 2022 and 31^st^ July 2024 after obtaining ethical approval from Institutional Review Committee (Reference number: IRC-LMC 05/S-22). All patients who underwent appendectomy within 24 months period were included in the study after obtaining informed consent. Negative appendectomy, autolyzed specimen, and clinically suspected neoplasms were excluded from the study. Diagnosis of presumed appendicitis was made based on the clinical-radiological findings by the treating clinicians. Histopathologically, acute appendicitis is defined as neutrophilic infiltration within the appendiceal muscularis propria.^6^ Incidental neoplasm was defined as clinically unsuspected neoplasms which is identified incidentally only during pathological examination of resected specimen^3^. Negative appendectomy was defined as surgically removed appendix for clinically confirmed appendicitis with no inflammation on histopathology.^7^

Appendectomy specimens were received from the surgery department and were fixed in 10% buffered formalin solutions for 24 hours. A gross examination was done for mass, nodule, luminal content, perforation, and evidence of inflammation. Representative longitudinal sections from the tip and transverse sections were submitted from the body and base of the vermiform appendix for tissue processing. Tissue was processed in the automated tissue processor Leica TP1020. Sections were embedded in paraffin wax; the tissue block was sectioned at 3-4 microns, and stained with hematoxylin and eosin. Sections were subjected to microscopic examination and detailed morphologic features were recorded. They were seen under the Magnus MX21iLED microscope. Extra sections were re-grossed, prepared, and examined in cases of non-inflamed, suspected granulomatous, or tumoral appendices. Results were analyzed using IBM SPSS Statistics for Windows, version 27 (IBM Corp., Armonk, N.Y., USA). Mean, median, and standard deviation were calculated for numeric values. Frequency/percentage tables were created.

## RESULTS

A total of 350 specimens of appendices were received in the histopathology department during a 24-month period, out of which 30 (8.57%) specimens were excluded for negative appendectomies. Among the negative appendectomies, female were 18 (60%) and male were 12 (40%). The final sample size for the study was 320 out of which, there were 183 (57.19%) male and 137 (42.81%) female. The median age was 26 years (IQR: 20-35 years). There were 262 (81.87%) cases of appendicitis occurring before 40 years of age (Table 1).

**Table 1:** Age group and gender distribution of patients with clinic-radiological diagnosis of acute appendicitis (n=320).

Age Group	Male (n=183)	Female (n=137)	Total (n=320)
<10 Yr	5 (1.56)	5 (1.56)	10 (3.12)
10-19 Yr	38 (11.88)	30 (9.37)	68 (21.25)
20-29 Yr	65 (20.31)	51 (15.94)	116 (36.25)
30-39 Yr	37 (11.56)	31 (9.69)	68 (21.25)
40-49 Yr	19 (5.94)	9 (2.81)	28 (8.75)
50-59 Yr	11 (3.44)	7 (2.19)	18 (5.63)
>60 Yr	8 (2.50)	4 (1.25)	12 (3.75)
Total	183 (57.19)	137 (42.81)	320 (100)

The histopathological diagnosis of acute appendicitis was seen in 164 (51.25%) of cases (Table 2). Fecolith was identified in 118 (36.87%), perforation in 15 (4.68%), granulomatous appendicitis and lymphoid hyperplasia in 3 (0.94%) each while neurogenic appendicopathy and *Enterobious vermicularis* in 1 (0.31%) each.

**Table 2:** Histopathological findings of the appendix operated for the clinic-radiological diagnosis of acute appendicitis (n=320).

Diagnosis	n (%)
Acute appendicitis	164 (51.25)
Acute suppurative appendicitis	148 (46.25)
Gangrenous appendicitis	38 (11.87)
Periappendicitis	15 (4.68)

A total of 6 (1.87%) incidental neoplasms were identified out of which 3 (0.94%) were carcinoid tumors. All 3 carcinoid tumors were identified at the tip of the appendix. The age of presentation of adenocarcinoma was more than 60 years, Low Grade Appendiceal Mucinous Neoplasm (LAMN) was 58 years, carcinoid was 35 years and male to female ration was 2.1 (Table 3).

**Table 3:** Age group and gender distribution of patients with clinic-radiological diagnosis of acute appendicitis (n=320).

Incidental neoplasms	n (%)	Male n(%)	Female n(%)
Carcinoid	3 (0.94)	2 (0.62)	1 (0.31)
LAMN	1 (0.31)	1 (0.31)	0 (0)
Adenocarcinoma NOS	2 (0.62)	1 (0.31)	1 (0.31)
Total	6 (1.87)	4 (1.25)	2 (0.62)

LAMN: Low-Grade Appendiceal Mucinous Neoplasm; NOS: Not Specified Otherwise

## DISCUSSION

As appendicitis is a common surgico-pathological disease that poses a great health burden to a large population. It is essential to thoroughly examine appendectomy specimens.^8,9^. Out of 350 total appendectomy cases, all of which were clinically and radiologically diagnosed as acute appendicitis, only 320 had positive findings in the appendix, 6 (1.87%) of which were incidental neoplasms.

There were 30 (8.57%) cases of negative appendectomy in our study, which is higher than the study done by Abd al-Fatah et al.^10^ where only 5% of appendectomy specimens had negative appendectomies, but this was similar to a study done by Shrestha et al.^11^ which showed 10.8% with negative appendectomies. A study done by Jones et al.^12^ had negative appendectomies in 23% of laparoscopically removed appendices. According to Choi et. al^13^, a 15.25% of negative appendectomy rate is acceptable to avoid the risk of complications due to delayed or missed diagnosis of potential life-threating complications.

In our study, a significantly, higher number of females had negative appendectomies. This was concordant with the study by Shrestha et al.^11^ which showed females with significantly higher negative appendectomies (p<0.007). A similar male to female ratio was seen in other studies.^10,14,15^ Negative appendectomy is a relatively common surgical issue with multiple risk factors contributing to it, including female gender and various clinical and radiological mimickers of appendicitis.^12,13^

The gender distribution in our study suggests a male predominance with 183(57.19%) male and 137(42.81%) female, and M:F ratio of 1.3:1. This was concordant with other studies that had similar male to female ratio. ^8-10,16-19^. However, this data is discordant with another study by Menyangbo et. al^2^, where M:F was 0.7:1. Another study by Sona R. et al^20^ shows an even higher male to female ratio of 2.8:1.

The mean age of presentation in our study was 28.73 years with an age range of 6-74 years, peaking at 20-29 years. More than 80% were diagnosed before 40 years. A similar mean age of presentation was seen in several studies. ^2,10,17,18^ A majority of cases ranging from 7085% are diagnosed under the age of 40 years.^9,18^ The peak incidence for most studies was between the ages of 20-29 years of age.^2,8,10,18,19^ However, a different peak of 11-20years was seen in Poudel et al.^16^

Histopathological diagnosis of acute appendicitis in our study was 164 (51.25)%, Acute suppurative appendicitis was 148 (46.25)%, and gangrenous appendicitis was 38 (11.87%). These complications indicate clinical progression of acute appendicitis. These values are heterogeneous across various studies with acute appendicitis being the commonest, diagnosed in 45.5%, 53%, and 34.75% of appendectomy specimens respectively, closely followed by acute suppurative appendicitis.^9,10,19^ Gangrenous appendicitis is 38 (11.87%) and perforation of 15 (4.68%) was seen in our study. Potey et al.^21^ showed a high (13.8%) number of gangrenous appendicitis with perforation. In MyAgeri et al.^6^, perforation was seen in 39.6% of appendices. However, they also reported that only 27.5% of patients reported within the first 24 hours of clinical presentation. Poudel et al.^16^ and AlQahtani et al.^17^, was concordant with our study with gangrenous appendicitis being diagnosed in 16.3% of cases and 10% respectively. These inflammatory appendiceal complications indicates delay in presentation and obstructive luminal pathology, such as faecolith, parasites, lymphoid hyperplasia. In our study fecolith was identified in most cases with acute appendicitis 118 (36.87%).^6^

Incidental finding of Enterobious vermicularis was seen in 1(0.31)% cases in our study and many other studies also found this parasite on histopathological examination, with incidences of 1.1%, 0.7%, 0.63 and 0.8% showing concordance with our study as parasitic appendicitis is invariably an incidental findings without clinical-radiological suspicion.^9,12,16,18,20^ However, study done by Abd al-Fatah et al.^10^, showed a very high incidence of 4%. The space occupying appendiceal neoplasm causing obstruction contributes to clinical features of acute appendicitis at presentation.

Many appendiceal masses are found incidentally during pathological workups for presumed appendicitis. This study focuses on the histopathological workup and possible identification of incidental pathology. Carcinoid is a rare, slow-growing, neuroendocrine tumour usually located at the tip of the appendix as a tiny radiologically inapparent nodule. Out of 320 cases, 6 (1.87) neoplasms were diagnosed incidentally in our study, of which carcinoids were diagnosed in 3 (0.94%) cases, Adenocarcinoma NOS in 2 (0.62%) cases and LAMN in 1 (0.3%) case. All had clinical and radiological suspicion of acute appendicitis or appendicular lump. Acute neoplasm is rare, many of which are occult in radiology. When faced with unusual presentation of acute appendicitis, it is crucial for radiologist to consider appendicular neoplasia.^3,22^ The incidence of neoplasia in Rencuzogullari et al,^4^ shows concordance with our study showing 2.6% of all diagnoses.

Carcinoid was seen in 3(0.9%) with male to female ratio of 2:1 and mean age of presentation of 32.33 years. Carcinoid is relatively rare, with its diagnosis seen in 0.43%, 0.77%, 0.88%, 0.19%, and 1.06% of appendectomy specimens across various studies showing concordance with our study.^4,9,12,18,19^ Furthermore, carcinoid discovered were located within submucosa in this study and muscularis propria extension, mesoappendix and angioinvasion were not seen. Study by Abd al-Fatah et al.^10^ shows discordance with our study due to high incidence of carcinoids (2.17%) in their study. The median age of presentation of neuroendocrine tumor is 55.5 years in Rencuzgullari et al^4^, not matching our study. While, in Barut et al.^22^, the median age was 37.5 years, showing younger age of presentation which was similar with this study. Male to female ratio in this study also shows a high male to female ratio of 1.5:1. Rencuzgullari et al.^4^ also had gender distribution similar to ours with Male: Female ratio of 1.75:1 for carcinoid tumors.

This study has several limitations that need to be considered. The cross-sectional design limits the ability to establish causal relationships. The study was conducted in a single tertiary care centre, which may restrict the applicability of findings to other populations with different demographic and clinical characteristics. Molecular and immunohistochemical analyses, were not included in the tumors encountered, due to non availability in our institution, which could have provided more detailed insights into the sub-type and prognosis of the disease.

## CONCLUSIONS

The histopathological spectrum of suspected acute appendicitis in appendectomy consisted of acute, suppurative and gangrenous appendicitis. Appendiceal neoplasms are uncommon which was comparable to other previous studies. Carcinoid tumor was the most common incidental neoplasm.
